# Having a Casino in the Community: Implications for Older Residents

**DOI:** 10.1093/geroni/igaa057.174

**Published:** 2020-12-16

**Authors:** Cindy Bui, Caitlin Coyle

**Affiliations:** University of Massachusetts Boston, Boston, Massachusetts, United States

## Abstract

Casino-going has been acknowledged as a common leisure activity for older adults, but what having a casino in the community means for the local older population has been understudied. Previous research has focused on problem gambling among older adults, but little is understood about how older residents perceive having a casino nearby and further how it impacts relevant senior services. This mixed-methods study gathered perspectives from 14 senior center directors and older residents (N = 411) of communities in Massachusetts that surround Plainridge Park Casino, the first casino that opened in the state in 2015. We conducted qualitative interviews with senior center directors and distributed a quantitative survey to older residents of the surrounding communities and those who visited the casino during the study period. We found that while most senior centers did not engage in trips to this “hometown” casino, many had other creative interactions with the casino, such as using casino space to host senior center events or seeking funding support from the casino. Older residents exhibited low rates of problem gambling risk, preferred to go to casinos outside of the state as an excursion, and attributed their reasoning to go to casinos to socializing rather than gambling aspects. While we must be aware of risks for problem gambling, this study also highlights the possibility of reframing a local casino as a potential asset that could contribute back to the community by providing resources for senior service providers and expanding social engagement opportunities for the older population.

